# Langerhans Cell Sarcoma of the Axillary Lymph Node: A Case Report and Review of the Literature

**DOI:** 10.5152/tjh.2011.98

**Published:** 2013-06-05

**Authors:** Aylin Orgen Çallı, Yelda Morgül, İnci Alacacıoğlu, Sadi Bener, Bahriye Payzin

**Affiliations:** 1 İzmir Atatürk Training and Research Hospital, Department of Pathology, İzmir, Turkey; 2 İzmir Training and Research Hospital, Department of Hematology, İzmir, Turkey

**Keywords:** Langerhans, Sarcoma, Axillary, Lymph node, Differential diagnosis

## Abstract

Langerhans cell sarcoma is a rare, high-grade neoplasm with overtly malignant cytological features and the Langerhans phenotype. Herein, we present a rare case of Langerhans cell sarcoma in a 65-year-old female that presented with a painless enlarging mass in her right axillary region, along with the histopathological features and diagnostic characteristics in the light of literature on Langerhans cell sarcoma.

## Abstract (tr)

Langerhans hücreli sarkom malign sitolojik görünümlü ve Langerhans fenotipine sahip nadir görülen yüksek dereceli neoplazmdır. Biz, sağ aksiller bölgede büyümüş ağrısız kitlesi olan 65 yaşında Langerhans hücreli sarkom olgusunu sunduk ve Langerhans hücreli sarkomun tanısal ve histopatolojik özelliklerini literatür eşliğinde gözden geçirdik.

## INTRODUCTION

Langerhans cell sarcoma (LCS), also known as malignant histiocytosis X, is a rare entity defined as a tumor characterized by its LC immunophenotype and appearance. It has a high mitotic rate and anaplastic cytological features, and is usually observed in lymph nodes and skin, but may also affect the liver, spleen, and lungs [1,2,3,4,5]. The immunoprofile is (CD1a+, S100+), and some cells should have the characteristic LC features of grooved nuclei and/or Birbeck granules. LCS tends to affect the elderly and has an aggressive clinical course associated with a high mortality rate despite aggressive treatment [4].

Herein we report a new and unusual case of LCS in a 65-year-old female that presented with a mass in her axillary lymph node. The aim of this report was to emphasize that LCS, although rare, should be considered in the differential diagnosis because of its ability to mimic metastatic lesions in lymph nodes.

## CASE PRESENTATION

A 66-year-old female presented with a mass in the axilla that had been present for 4-5 months. Neither systemic symptoms nor a positive family history was observed. The patient had a history of type-2 diabetes mellitus and hypertension, and was using oral antidiabetics and amlodipine. All laboratory tests (complete blood count, serology, and microbiology), ultrasonographic examination, and computed tomography of the head and neck region, lungs, and lower abdomen were normal. At another clinic the mass was excised and diagnosed as breast carcinoma. The patient was referred to our hospital for further investigation and treatment. Paraffin blocks were sent to our department for further pathological examination.

**Figure 1:**
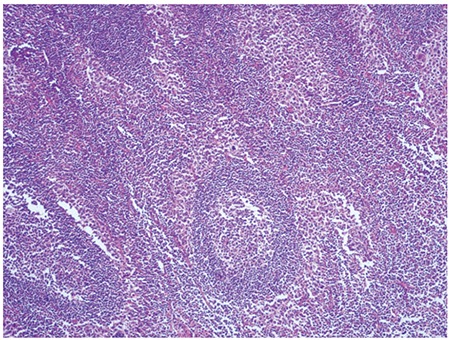
Low power view of LCS (HEx100).

**Figure 2:**
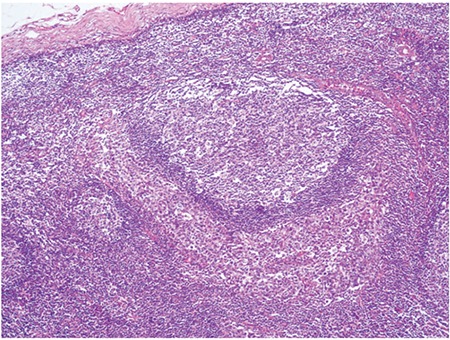
The follicle surrounded large, pleomorphic cells(HEx100).

**Figure 3:**
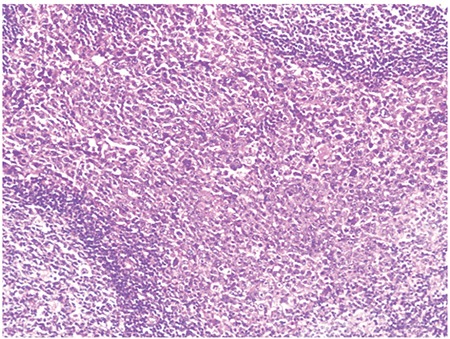
Hematoxylin and eosin staining showing lymph node involvement of langerhans cell carcinoma. Sinusoidal infiltration by lymphoma cells is evident (HEx200).

**Figure 4:**
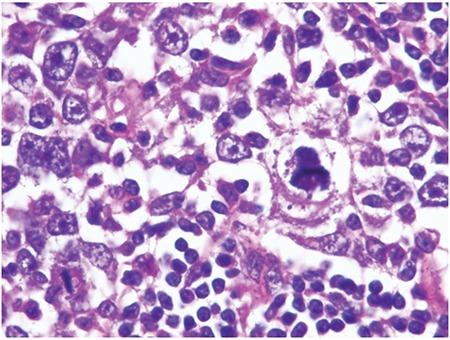
Histopathologic slides shows large atypical cells with malignant features including hyperchromatic nuclei with prominent nucleoli, and high mitotic rate (HEx400).

**Figure 5:**
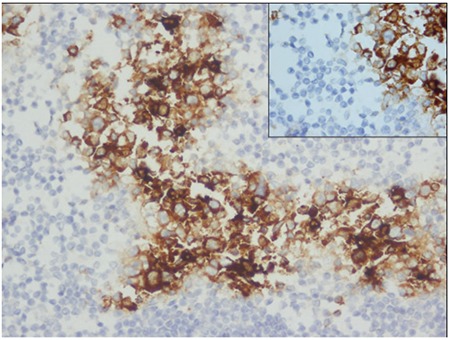
Tumor cells diffuse positive for immunostain for CD1a (x200).

**Figure 6:**
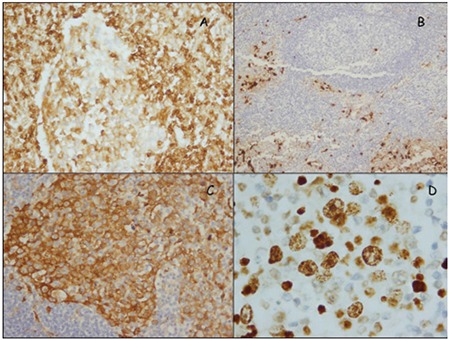
Tumor cells positive for immunostain for LCA (x400) (A), Tumor cells positive for immunostain for S-100 (x200) (B), Tumor cells positive for immunostain for EMA x400) (C), Tumor cells showed high Ki-67 proliferation index (x1000) (D).

Microscopic observation showed that the lymph node architecture was partially preserved, with retention of normal or hypoplastic germinal centers (Figure 1). The infiltrate was seen primarily in the parafollicular and subcapsular regions (Figure 2). The patient’s sinuses were distended by large and pleomorphic tumor cells that contained abundant pale eosinophilic cytoplasm and bizarre, grooved nuclei (Figure 3). Many of the cells exhibited multinucleation, nuclear lobulation, and high mitotic activity (Figure 4). The neoplastic cells had the LC immunophenotype, and strongly expressed CD1a and S-100 (Figure 5, 6). The cells were also positive for fascin and weakly positive for CD45. The tumor cells were negative for CD30, ALK-1, CD-2, CD-3, CD-4, CD-8, CD-7, CD-11c, CD-20, CD-21, CD-45 RO, CD-68, granzyme B, Bcl-2 protein, HMB-45, Melan A, EMA, pan cytokeratin, and follicular dendritic cell marker (CD21). The biopsy specimen was diagnosed as LCS. The patient underwent total surgical resection. No chemotherapy or radiotherapy was planned. At the 1-year follow-up the patient was doing well.

## DISCUSSION

Mature/fully-differentiated histiocytic/dendritic cell neoplasms rarely affect hematopoietic or lymphoid tissues. They have been categorized into subtypes based on their location, enzyme histochemistry, ultrastructure, and immunohistochemical features, and are further classified into 5 groups designated by the WHO, as follows: LC histiocytosis (LCH); LCS; interdigitating dendritic cell sarcoma/tumor (IDCS/T); follicular dendritic cell sarcoma/tumor (FDCS/T); dendritic cell sarcoma, not otherwise specified [6].

The diagnosis of LCS is based on the following: malignant cytological features, such as atypia, hyperchromatic nuclei, prominent nucleoli, and frequent mitotic figures; the appearance of typical Birbeck granules; a typical immunophenotype with consistent expression of CD1a and S-100 protein, and langerin. The most helpful clues in the diagnosis of LCS are the expression of several histiocytic markers, such as CD68, and the weak expression of lysozyme. As LCH is also a proliferating disorder of Langerhans cells, LCH stains positive for CD1a and S-100 protein, and at times exhibits Birbeck granules, as does LCS. Polylobated and multinucleated Langerhans cells are, however, observed in LCS, and their nuclei are cytologically benign. With approximately 10-20 mitoses 10 HPFs–1, mitotic activity is generally low. In contrast, LCS is often characterized by a preponderance of large cells with complex nuclear outlines and a substantial quantity of cytoplasm. The nuclei of tumor cells exhibit malignant cytological features, such as prominent nucleoli with occasional grooves and high mitotic activity [3,4,5,6].

LCS must be differentiated from histiocytic sarcoma and other dendritic cell tumors. Immunohistochemical studies are essential for the differential diagnosis, and neoplastic cells in LCS should stain with CD1a. CD1a is a highly sensitive and specific marker of LCS, as compared to other dendritic cells and dendritic cell neoplasms [7] (Table 1). LCS is an extremely rare aggressive neoplasm that occurs in lymph nodes and extranodal sites, such as the skin, gall bladder, and bone [1,2,3,4,5]. To the best of our knowledge the English-language literature includes only 25 previously reported cases of LCS (Table 2) [1,2,3,4,5,6,8,9,10,11,12,13,14,15].

LCS is usually associated with multiple organ involvement, including the skin, lymph nodes, lungs, bone, spleen, and liver. Patients range in age between 10 and 81 years, with a male-female ratio of approximately 1:1. At present, there are no reports on the organized series of treatment for LCS. The majority of reported cases had a short survival time and a poor prognosis, as shown in Table 1; 50% (12 patients) of 24 patients died of the disease within 2 years. Successful treatment of advanced multiple organ diseases is achievable with such systemic combination chemotherapy as the CHOP regimen (cyclophosphamide-doxorubicin hydrochloride-vincristine-prednisolone), which was used in 15 of the published cases [16]. When considering the findings associated with localized LCS, radiotherapy might be the best treatment; however, many more LCS case findings are necessary to more clearly identify an optimal treatment strategy. The presented case was treated with total excision of the mass only, and did not receive adjuvant chemotherapy or radiation. The patient was doing well at the 1-year follow-up.

In conclusion, LCS of the axillary lymph node is a rare lesion that mimics metastasis of breast cancer, both clinically and radiologically. Correct diagnosis can only be made based on histopathological examination. Due to the vastly different treatment options, pathologists should be aware of this unusual neoplasm in order to facilitate correct diagnosis.

## Figures and Tables

**Table 1:**
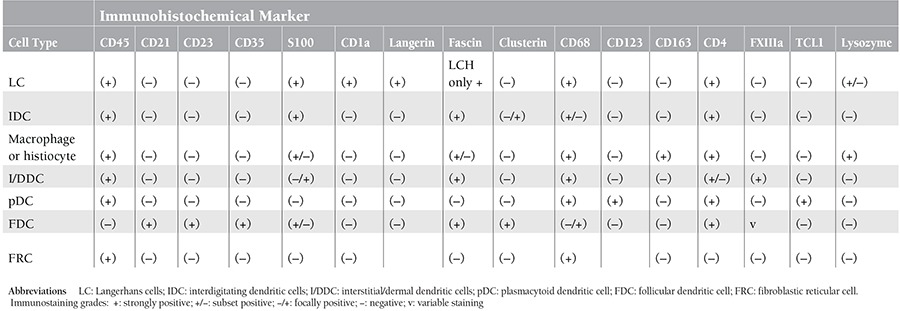
Immunohistochemical profile of dendritic, histiocyte, and stromal cell types.

**Table 2:**
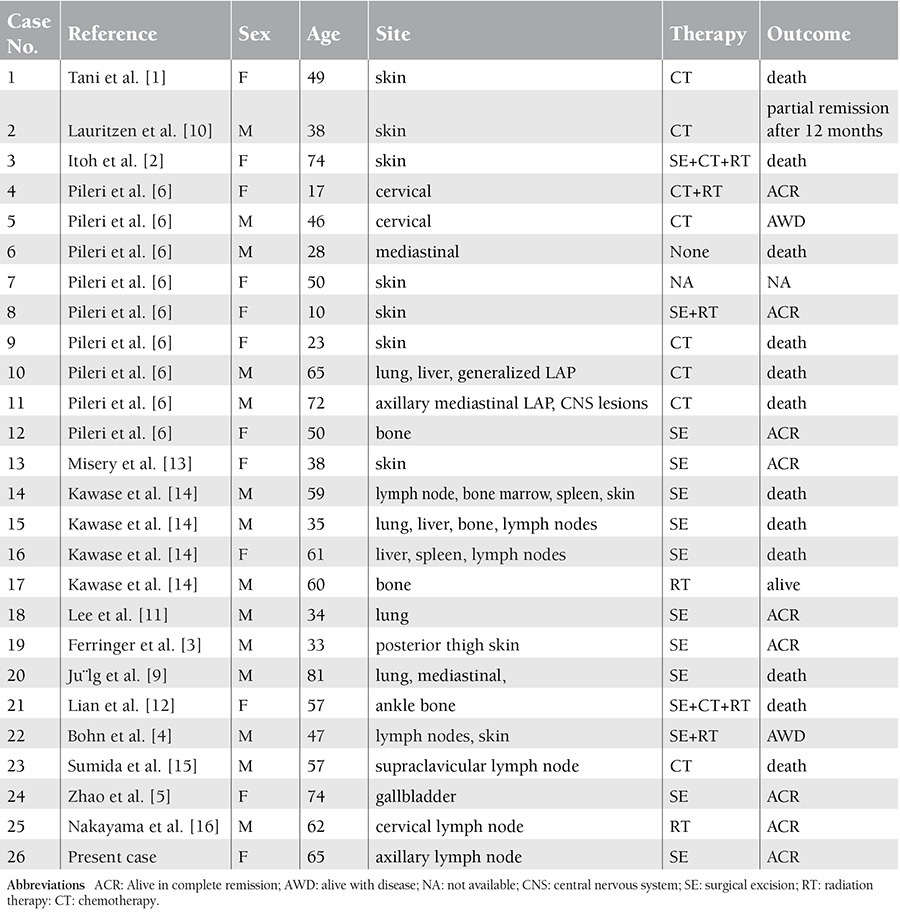
LCS cases reported since 1992.
